# The need for integrated public health surveillance to address sexually transmitted and blood-borne syndemics

**DOI:** 10.14745/ccdr.v45i23a03

**Published:** 2019-02-07

**Authors:** M Murti, J Wong, M Whelan, C Renda, K Hohenadel, L Macdonald, D Parry

**Affiliations:** 1 Public Health Ontario, Toronto, ON; 2 Dalla Lana School of Public Health, University of Toronto, Toronto, ON; 3 British Columbia Centre for Disease Control, Vancouver, BC; 4 School of Population and Public Health, University of British Columbia, Vancouver, BC

**Keywords:** surveillance, syndemic, STI, blood-borne infections, data integration, STBBI

## Abstract

A national approach to addressing sexually transmitted and blood-borne infections (STBBIs) was recently articulated in the Public Health Agency of Canada’s new *A Pan-Canadian Framework for Action: Reducing the health impact of sexually transmitted and blood-borne infections in Canada by 2030*. This Framework promotes an integrated approach, with a focus on the key populations that are affected by overlapping epidemics (i.e., syndemics). We advance the idea that integrating surveillance would be helpful in characterizing and understanding the populations, locations, risk behaviours and other drivers that contribute to STBBI syndemics. The creation of matched or linked data systems that would allow routine reporting of integrated data is challenged by the technical barriers of integrating data silos as well as by the privacy and ethical considerations of merging sensitive individual-level data. Lessons can be learned from jurisdictions where an improved understanding of syndemics, through integrated STBBI surveillance, has led to more efficient and effective operational, program and policy decisions. Emerging enablers include the development of data standards and guidelines, investment in resources to overcome technical challenges and community engagement to support the ethical and non-stigmatizing use of integrated data. The Framework’s call to action offers an opportunity for national discussion on priorities and resources needed to advance STBBI syndemic surveillance for local, regional and national reporting in Canada.

## Introduction

We applaud the Public Health Agency’s new document, *A Pan-Canadian Framework for Action: Reducing the health impact of sexually transmitted and blood-borne infections in Canada by 2030*, as a roadmap for federal, provincial/territorial and local public health jurisdictions to consider in their efforts to reduce the health impacts of sexually transmitted and blood-borne infections (STBBIs) in Canada (1). One of the Framework’s guiding principles is to move beyond programs and interventions targeted towards single infections by utilizing integrated approaches designed to “address the complexity and interrelated nature of risk factors and transmission routes for STBBIs”—that is, a syndemics approach.

Merrill Singer, a noted medical anthropologist, defined syndemics as “two or more health issues [that] interact synergistically to contribute to increased health burden for individuals or communities” (2). Singer used the interaction of human immunodeficiency virus (HIV) and tuberculosis (TB) co-infections to typify the adverse interactions between two diseases (i.e., the more rapid progression of symptoms and illness occurring from concurrent infections versus single infections of either), and the common conditions of health inequity (e.g., poverty and marginalization) that contribute to their clustering and spread (3). In a more recent example, a study from British Columbia characterized the epidemic of syphilis among gay, bisexual and other men who have sex with men, and its associations with substance use, mental health and co-infection with HIV. Additionally, the association was higher for those reporting multiple co-morbid conditions (4). Applying a “syndemic lens” enhances efforts to measure and address known risk factors for single diseases by characterizing their multiplicative effects and impacts across diseases. Syndemics also necessitates community engagement and ethical frameworks to ensure that data are presented in such a way as to prevent further stigmatizing of vulnerable groups that may be associated with an excess burden of illness.

The goals of the Framework would be well supported by public health surveillance that uses an integrated approach to help inform and prioritize public health action. Currently, most public health surveillance systems focus on single diseases. They are not designed to report on multiple and interacting causes of STBBIs, such as concurrent and previous STBBIs, common risk factors across infections, social determinants of health and environmental factors (e.g., availability of testing/treatment locations). Understanding the synergistic impacts of these causes of disease would enable the development and prioritization of strategic operational, program and policy decisions (5). For example, monitoring the timing and prevalence of sexually transmitted co-infections, along with factors impacting testing and treatment, could be used in the planning and evaluation of HIV preexposure prophylaxis programs, particularly as these programs are being scaled up across Canada (6).

Although an integrated STBBI surveillance system is a laudable goal, creating the necessary data infrastructure and supporting policies is a major undertaking. The objective of this article is to highlight examples of jurisdictions currently engaged in this work and to explore the challenges and potential enablers to adopting integrated STBBI surveillance systems.

## Examples of surveillance integration

Public health organizations around the world are engaged in a range of activities to integrate and report on STBBI syndemics. An example of this activity is AtlasPlus: an interactive website, hosted by the United States’ Centers for Disease Control and Prevention (CDC), designed to visualize the rates of STBBIs and TB infections and the measures of social determinants of health at the state level (7). AtlasPlus enables side-by-side comparisons of combinations of metrics for two infections, along with area-based social determinants, but does not include linked or matched data. There are two significant advantages of this tool: single-disease data can be refreshed independently and in a timely manner; and any number of measures of social determinants or other drivers can be overlaid as long as they are available at similar geographic levels. While interpretations are limited to ecological associations, this tool stimulates hypothesis-generation and further exploration.

The CDC has also supported the integration of surveillance for HIV, sexually transmitted infections (STIs), viral hepatitis and TB at both the state and local levels through two funding initiatives: the Outcome Assessment through Systems of Integrated Surveillance (OASIS) initiated in 1998; and the Program Collaboration and Service Integration initiated in 2007 (8,9). These initiatives provided funding for database development and established communities of practice to advance capacity-building for the analysis and use of integrated data. The New York City Health Department created a 10-year matched dataset of patients with viral hepatitis, TB, STIs and HIV, complete with vital statistics death data. With this cohort, they described the overlapping risk factors across infections and higher than expected mortality associated with multiple infections (10). Subsequently, non-infectious disease outcomes, such as the prevalence of diabetes, are now being matched to this dataset to examine their associations with STBBIs and TB (11).

Another example is from Los Angeles County Public Health, where neighbourhood maps were overlaid with information on HIV/STI co-infection clusters, locations of existing testing sites and income measures to identify high burden service planning areas (12). Los Angeles County Public Health have gone on to develop health district profile maps, using matched data, that allow for reporting of sociodemographics, behaviours, co-infections and recurrent infections at the level of individual patients. Demographic subgroup and infection characteristic data are helpful for targeting of programs. Health districts are also ranked by these indicators to support the geographic prioritization of resources for HIV and STI prevention and treatment (13).

Jurisdictions in Canada have similarly leveraged existing linked databases to assess the impact of co-infections in individuals. Alberta’s *2013 Annual Report on STI and HIV* included a matched analysis of the prevalence and timing of STI co-infection (before, same time or after HIV infection) in individuals diagnosed with HIV between 2005 and 2013 (14). These results demonstrated a change over time: before 2007, the majority of individuals were diagnosed with an STI before or at the same time as their HIV diagnosis, whereas in 2013, individuals were twice as likely to have been diagnosed with an STI after their HIV diagnosis. While the co-occurrence of HIV and STIs is well documented, this kind of surveillance data helped identify a local change in trends over time. This type of information can be used to guide STBBI programs to adapt their testing and prevention strategies for individuals who are at risk of, or who have been diagnosed with HIV.

An example of a jurisdiction moving from studies of one-time cohorts towards ongoing, integrated surveillance is the British Columbia Hepatitis Testers Cohort (BC-HTC). This is a population-based research cohort, using provincial STBBI surveillance and public health laboratory data that is linked to the BC Cancer Registry and BC’s prescription dispensing and healthcare utilization databases. This cohort has been used to monitor a cascade of care for hepatitis C within key populations, including people living with HIV and people who inject drugs (15). Because this cohort is updated regularly, ongoing cohort analyses are expected to help inform the development and evaluation of programs and policies addressing this syndemic (16). As well, the matching algorithms developed for the BC-HTC are now being applied to improve routine surveillance from the BC Centre for Disease Control data warehouse that facilitates STBBI reporting for BC (17).

## Challenges and enablers

For both BC and New York City, many years were required to create the infrastructure needed to analyze key populations. In light of this, other jurisdictions that may not have begun this process may wonder whether the questions answered by integrated STBBI data are worth the efforts, compared with what can be learned when traditional epidemiological methods are applied to single diseases. Even if there is support for integrated surveillance, there may still be debate on:

- What are the most important questions that need to be answered by integrated surveillance?
- What is the most appropriate level (local, regional, national) to address those questions?

Jurisdictions that already have the infrastructure in place may now face the challenges of working with various stakeholders to identify priorities for outputs, when so many analyses are now possible. Here, resource limitations may drive the prioritization of surveillance activities and, simultaneously, integrated surveillance can help guide the appropriate allocation of resources.

There are a number of challenges to integrated surveillance. In a 2007 survey of CDC-funded STI programs that examined the extent of surveillance integration in these programs (18), barriers to increased integration were first identified and included the following: restrictive data policies, particularly for HIV data that are siloed from other systems; incompatible databases, which complicated the linking of data; lack of time and technical expertise in linking and managing data systems; and lack of financial resourcing. Subsequent to this survey, the CDC published guidelines for STBBI data security and confidentiality that set out data standards for the technical and privacy considerations of collating STBBI data at the local, state and national levels (19). They also set out program standards for CDC-funded public health organizations, based on 10 guiding principles for data collection, storage, sharing and use to ensure security and confidentiality and 32 standards addressing program policies and responsibilities, data collection and use, data sharing and release, physical security and electronic data security.

In addition to overcoming the technical barriers to integrated surveillance, local-level engagement with communities is critical to ensuring that information from such integration does not increase stigmatization of key populations. Advancements in STBBI testing, particularly phylogenetic analyses, has not only raised new opportunities for assessing syndemics but also new ethical and privacy challenges as increased granularity in transmission dynamics may increase the potential for stigmatizing individuals and specific groups (20). One approach to address these challenges is the Ontario HIV Epidemiology and Surveillance Initiative’s “Champions Committee”, which is comprised of various community members and persons with specific, relevant life experiences; these committees review all the Initiative’s HIV surveillance products (21).

## Discussion

The document, *A Pan-Canadian STBBI Framework for Action,* calls for surveillance that supports integrated approaches to key populations affected by STBBI syndemics. To achieve these goals, it would be useful to characterize and monitor populations affected by STBBI co-infections and the various factors associated with these infections. While there are challenges and important ethical and privacy considerations for integrating these STBBI data, there are examples from leading jurisdictions, including some from within Canada, that demonstrate both that these challenges can be overcome and the benefits of doing so.

To realize the benefits of integration, organizations involved in STBBI surveillance will have to contend with the unique challenges of their information systems and their supporting data management and dissemination practices. These challenges can be addressed through funding initiatives, communities of practice addressing technical barriers, the development of white papers on data standards, and the establishment of frameworks for the ethical use of linked data.

### Conclusion

There is an urgent need to address the growing burden of STBBIs in Canada, and the recent Framework has created an agreed-upon national approach to do so. Ultimately, innovation, investment and collaboration will be needed to facilitate integration of STBBI surveillance to support the goals of this Framework.
